# Effect of Topical Antibiotics on Duration of Acute Infective Conjunctivitis in Children: A Randomized Clinical Trial and a Systematic Review and Meta-analysis

**DOI:** 10.1001/jamanetworkopen.2022.34459

**Published:** 2022-10-04

**Authors:** Minna Honkila, Ulla Koskela, Tero Kontiokari, Marja-Leena Mattila, Aila Kristo, Raija Valtonen, Suvi Sarlin, Niko Paalanne, Irma Ikäheimo, Tytti Pokka, Matti Uhari, Marjo Renko, Terhi Tapiainen

**Affiliations:** 1 Department of Pediatrics and Adolescent Medicine, Oulu University Hospital, Oulu, Finland; 2 Research Unit of Clinical Medicine and Medical Research Center Oulu, University of Oulu, Oulu, Finland; 3 Children’s Mehiläinen, Oulu, Finland; 4 Children’s Terveystalo, Oulu, Finland; 5 NordLab Oulu, Oulu, Finland; 6 University of Eastern Finland and Kuopio University Hospital, Kuopio, Finland; 7 Biocenter Oulu, University of Oulu, Oulu, Finland

## Abstract

**Importance:**

Although topical antibiotics are often prescribed for treating acute infective conjunctivitis in children, their efficacy is uncertain.

**Objective:**

To assess the efficacy of topical antibiotic therapy for acute infective conjunctivitis.

**Design, Setting, and Participants:**

A randomized clinical trial was conducted in primary health care in Oulu, Finland, from October 15, 2014, to February 7, 2020. Children aged 6 months to 7 years with acute infective conjunctivitis were eligible for enrollment. The participants were followed up for 14 days. A subsequent meta-analysis included the present trial and 3 previous randomized clinical trials enrolling pediatric patients aged 1 month to 18 years with acute infective conjunctivitis.

**Interventions:**

Participants in the present randomized clinical trial were randomized to moxifloxacin eye drops, placebo eye drops, or no intervention.

**Main Outcomes and Measures:**

The primary outcome in the present randomized clinical trial was time to clinical cure (in days); in the meta-analysis, the primary outcome was the proportion of participants with conjunctival symptoms on days 3 to 6.

**Results:**

The randomized clinical trial included 88 participants (46 [52%] girls), of whom 30 were randomized to moxifloxacin eye drops (mean [SD] age, 2.8 [1.6] years), 27 to placebo eye drops (mean [SD], age 3.0 [1.3] years), and 31 to no intervention (mean [SD] age, 3.2 [1.8] years). The time to clinical cure was significantly shorter in the moxifloxacin eye drop group than in the no intervention group (3.8 vs 5.7 days; difference, −1.9 days; 95% CI, −3.7 to −0.1 days; *P* = .04), while in the survival analysis both moxifloxacin and placebo eye drops significantly shortened the time to clinical cure relative to no intervention. In the meta-analysis, a total of 584 children were randomized (300 to topical antibiotics and 284 to a placebo), and the use of topical antibiotics was associated with a significant reduction in the proportion of children who had symptoms of conjunctivitis on days 3 to 6 compared with placebo eye drops (odds ratio, 0.59; 95% CI, 0.39 to 0.91).

**Conclusions and Relevance:**

In this randomized clinical trial and systematic review and meta-analysis, topical antibiotics were associated with significantly shorter durations of conjunctival symptoms in children with acute infective conjunctivitis.

**Trial Registration:**

ClinicalTrialsRegister.eu Identifier: 2013-005623-16

## Key Points

**Question:**

Are topical antibiotics effective for the management of acute infective conjunctivitis in children?

**Findings:**

In this randomized clinical trial with 88 participants, use of topical antibiotics reduced the time to clinical cure, and in the meta-analysis with 584 participants, the use of topical antibiotics was associated with a significant reduction in the proportion of children who had symptoms of conjunctivitis on days 3 to 6 compared with those receiving a placebo.

**Meaning:**

These findings suggest that topical antibiotic therapy should be considered for acute infective conjunctivitis in children because antibiotics were associated with significantly shorter recovery times.

## Teaser

This study assesses the efficacy of topical antibiotic therapy for acute infective conjunctivitis among children.

## Introduction

Acute conjunctivitis is a common infection in children and is usually caused by bacteria.^1^ Physicians often prescribe antibiotics for this ailment,^2^ although the evidence for their effectiveness in pediatric patients is limited and conflicting. Gigliotti et al^3^ reported in the first randomized trial of acute conjunctivitis in children that we know of that treatment with topical antibiotics resulted in a greater cure rate than that among those receiving placebo eye drops, whereas a trial conducted by Rose et al^4^ found that there was no difference in the clinical recovery between children treated with chloramphenicol eye drops and those treated with a placebo.^4^ However, since the placebo eye drops used by Rose et al^4^ contained antiseptic agents as preservatives, both groups may have received anti-infective therapy. Finally, to our knowledge, all previous meta-analyses showing modest beneficial effects of topical antibiotics have combined pediatric and adult patients.^5^

In this randomized clinical trial (RCT), children with acute infective conjunctivitis receiving topical antibiotics were compared with control participants receiving a placebo, while an additional control group that received no intervention was included because placebo eye drops alone may be beneficial due to their washout effect. The study was then continued by performing a systematic review and meta-analysis of data extracted from the present trial and previous RCTs.

## Methods

The protocol was found ethically acceptable by the Finnish Medical Agency and the Ethical Committee of the North Ostrobothnia’s Hospital District, Oulu, Finland. Only children whose parents or legal representatives provided written informed consent were included in the trial. The RCT followed the Consolidated Standards of Reporting Trials (CONSORT) guideline. The systematic review and meta-analysis were registered with Prospero (CRD42021255970) and were conducted in accordance with the Preferred Reporting Items for Systematic Reviews and Meta-analyses (PRISMA) guideline.^6^ The study protocol and statistical analysis plan are shown in Supplement 1.

### Trial Design

The RCT was first conducted to investigate the efficacy of moxifloxacin eye drops in the treatment of acute infective conjunctivitis in children compared with placebo and no intervention. Both placebo and no intervention controls were chosen to evaluate the potential washout effect of placebo eye drops. The trial was conducted at 2 pediatric outpatient clinics in the city of Oulu and at the pediatric emergency department of Oulu University Hospital, Oulu, Finland, from October 15, 2014, to February 7, 2020. Second, a systematic review and meta-analysis of the results of the present and previous RCTs was conducted to assess whether the evidence favors topical antibiotic therapy for the treatment of acute infective conjunctivitis.

### Participants, Randomization, and Blinding in the RCT

Children aged 6 months to 7 years with a clinical diagnosis of acute infective conjunctivitis were eligible for enrollment. Acute infective conjunctivitis was defined as the presence of conjunctival inflammation (erythema), discharge, or soreness or swelling of the eyelids. The exclusion criteria were allergy to fluoroquinolones, antibiotic therapy 7 days prior to the study, severe infection, allergic conjunctivitis, a trauma, or a foreign body in the eye. All the children were from Finnish-speaking families.

The eligible children were randomized to receive moxifloxacin eye drops, placebo eye drops, or no intervention in a 1:1:1 ratio. A biostatistician created a randomization list in permuted blocks of 6 in a random order, and the results were enclosed in sealed, numbered opaque envelopes containing either a code for the study group or the words no treatment. After informed consent had been obtained, the study physician opened the envelope and revealed a code (A or B) or no treatment.

The participating children, their parents, and the physicians and nurses were blinded to the assignment of the moxifloxacin and placebo eye drop groups, while the study design for the no intervention group was randomized but unblinded. The pharmacy distributed the eye drops and instructions to the families in opaque cardboard boxes in which the moxifloxacin eye drops were packed in plastic bottles and the placebo eye drops in single-dose vials. Both the bottles and the vials were transparent and did not have labels.

### RCT Procedures

Conjunctival and nasopharyngeal specimens subjected to respiratory pathogen polymerase chain reaction (PCR) testing and bacterial culture were obtained from the participants, and detailed descriptions of these microbiological samples are available in the study protocol (Supplement 1). The participants in the eye drop groups received either moxifloxacin eye drops (5 mg/mL [Novartis Finland]) or placebo eye drops (carboxymethylcellulose sodium, 1.0% [Allergan Pharmaceuticals Ireland]) administered as 1 drop in each affected eye 3 times daily until conjunctival symptoms were absent for at least 24 hours. The maximum duration of treatment was 7 days. The placebo eye drops used in the trial did not contain preservatives. All parents, including those in the no intervention group, were advised to remove any discharge from the child’s eyes at least 3 times a day. Moxifloxacin eye drops were started in a nonblinded manner as a rescue treatment if needed.

### Primary and Secondary Outcomes of the RCT

The primary outcome was time (days) to clinical cure, defined as resolution of all conjunctival symptoms without relapse for 2 days. The secondary outcome was a relapse of conjunctivitis within 14 days of randomization. The outcomes were identified from the daily symptom sheet diaries that the parents completed for 14 days. In addition, the physician contacted the parents after the 14-day follow-up to ensure a clinical cure.

### Systematic Review and Meta-analysis

RCTs were considered eligible if they compared topical or systemic antibiotics for the management of acute conjunctivitis in children and adolescents aged 1 month to 18 years with a control group without antibiotic therapy. We identified the literature in ClinicalTrials.gov, Cochrane Library, Google Scholar, PubMed, ScienceDirect, and Scopus from database inception up to November 9, 2021, using the Medical Subjects Headings terms *conjunctivitis, bacterial* NOT *trachoma* for the literature search. Two investigators (M.H. and U.K.) independently screened the titles and abstracts to identify eligible studies according to the given inclusion criteria. After that, 3 investigators (M.H., U.K., and T.T.) reviewed the full manuscripts to determine those to be included and excluded. Data for all variables were extracted independently by 3 investigators (M.H., U.K., and T.T.).

The primary outcome of the meta-analysis was the proportion of participants who had conjunctival symptoms on days 3 to 6, while secondary outcomes were the proportion of participants who had conjunctival symptoms on days 7 to 10 and the proportion without a microbiological cure on days 7 to 10. The trials were assessed for the risk of bias independently by 3 investigators (M.H., U.K., and T.T.) using the Cochrane Collaboration tool for assessing the risk of bias in randomized trials,^7^ and consensus was reached by discussion.

### Statistical Analysis

Based on prior studies,^4^ we estimated that acute infective conjunctivitis in children will resolve in a mean (SD) of 5 (2) days without treatment, so a 1.5-day difference in cure time could be considered clinically significant. With a statistical power of 80% and a 2-sided α error of .05, we calculated that the sample size needed was 29 children per group.

A 1-way analysis of variance test, followed by a Tukey post hoc test, was used to compare the mean times to clinical cure between the moxifloxacin, placebo, and no intervention groups with 95% CIs. Variations in the cure times were compared between the moxifloxacin group and both control groups separately with Kaplan-Meier log rank (Mantel-Cox) survival statistics. The χ^2^ test was used to test the statistical significances of the differences in relapse rates between the 3 groups. Data were analyzed with SPSS statistics software version 27.0 (IBM Corp).

Data from the RCTs in the meta-analysis were used to calculate odds ratios (ORs) and 95% CIs comparing the intervention with a placebo. The *I^2^* statistic was used to investigate the heterogeneity between the trials. A random-effects model was chosen for this, as it was assumed that the RCTs represented a random sample of all studies evaluating the efficacy of treatment for acute infective conjunctivitis. Potential publication bias in the trials was analyzed using a funnel plot and the Egger or Harbord-Egger test. The analyses of the outcomes were performed using Comprehensive Meta Analysis software version 3.3.070 (Biostat, Inc).

## Results

### Participants in the RCT

**Figure 1.**
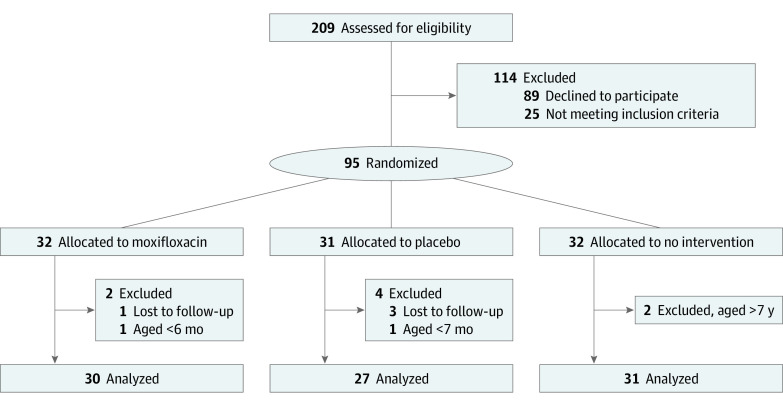
Flow Diagram of the Randomized Clinical Trial

A total of 209 children with acute conjunctivitis were evaluated for eligibility, and 114 children were excluded: 25 because they did not meet the inclusion criteria, and 89 because their families declined to participate (Figure 1). After randomization of the remaining 95 children, 4 children were lost to follow-up and 3 were excluded from the analyses because they did not meet the predefined age criteria. Altogether 30 children in the moxifloxacin group, 27 in the placebo group, and 31 in the no intervention group were included in the analyses for a total sample size of 88. There were 46 girls (52%). The mean (SD) age of the children was 2.8 (1.6) years in the moxifloxacin group, 3.0 (1.3) years in the placebo group and 3.2 (1.8) years in the no intervention group (eTable 1 in Supplement 2).

Most children had symptoms of respiratory tract infection and a positive nasopharyngeal PCR for respiratory viruses (eTables 1 and 2 in Supplement 2). Conjunctival specimens for respiratory viruses were positive in 5 of 28 children (17.9%) in the moxifloxacin group, 3 of 25 (12%) in the placebo group, and 2 of 31 (6.5%) in the no intervention group (eTable 2 in Supplement 2). Overall, 70 children (83.3%) had a positive bacterial conjunctival culture, and the most common conjunctival bacterial pathogen was *Haemophilus influenzae* in all groups.

### Outcomes of the Trial

**Figure 2.**
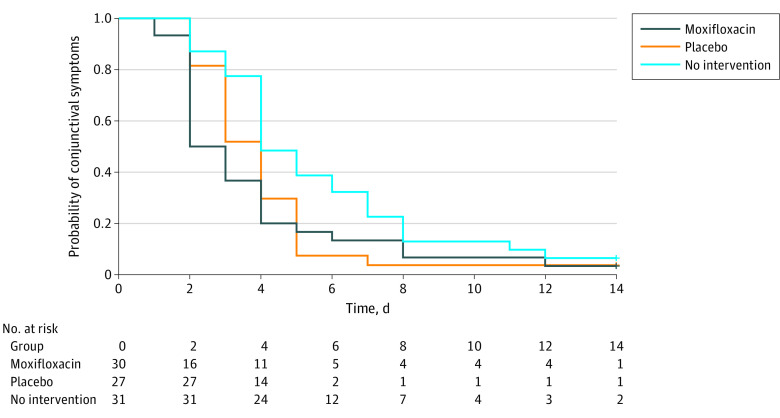
Probability of Conjunctival Symptoms in Children With Acute Infective Conjunctivitis Receiving Moxifloxacin Eye Drops, Placebo Eye Drops, or No Intervention Both moxifloxacin and placebo eye drops significantly shortened the time to clinical cure vs no intervention (*P* = .02 and *P* = .03, respectively).

The time to clinical cure was shorter in participants who received moxifloxacin eye drops than in those who did not receive any intervention (3.8 vs 5.7 days; difference, –1.9 days; 95% CI, –3.7 to –0.1 days; *P* = .04) (Table). When the children receiving moxifloxacin eye drops were compared with those receiving placebo eye drops, there was no difference in the time to clinical cure (3.8 vs 4.0 days; difference, 0.2 days; 95% CI, –2.2 to 1.6 days; *P* = .94). Similarly, when the children receiving placebo eye drops were compared with those who did not receive any intervention, there was no significant difference in the time to clinical cure (4.0 vs 5.7 days; difference, 1.7 days; 95% CI, –0.2 to 3.5 days; *P* = .10). In the Kaplan-Meier survival analysis, both moxifloxacin and placebo eye drops significantly shortened the time to clinical cure relative to no intervention (*P* = .02 and *P* = .03, respectively) (Figure 2).

**Table.** Trial Outcomes for 88 Participants Receiving Moxifloxacin Eye Drops Compared With Those Receiving Placebo Eye Drops and No Intervention

Outcome	Moxifloxacin (n = 30)	Placebo (n = 27)	No intervention (n = 31)	Moxifloxacin vs placebo		Moxifloxacin vs no intervention	
				Difference (95% CI)	*P* value	Difference (95% CI)	*P* value
Primary outcome							
Time to clinical cure, mean (SD), d	3.8 (3.1)	4.0 (2.3)	5.7 (3.3)	0.2 (–2.2 to 1.6)	.94^a^	–1.9 (–3.7 to –0.1)	.04^a^
Secondary outcome							
Relapse of conjunctivitis within 14 d, No. (%)	5 (17)	2 (7.4)	1 (3.2)	NA	.43^b^	NA	.10^b^

Abbreviation: NA, not applicable.

^a^ *P* value in a Tukey test performed after a one-way analysis of variance test (*P* = .03 in the 1-way analysis of variance).

^b^ *P* value in a χ^2^ test.

Relapse of conjunctivitis occurred in 5 children (17.9%) in the moxifloxacin group, 2 children (7.4%) in the placebo group, and 1 child (3.2%) in the no intervention group (*P* = .43 for moxifloxacin vs placebo eye drops and *P* = .10 for moxifloxacin eye drops vs no intervention) (Table). Moxifloxacin eye drops were started in a nonblinded manner as a rescue treatment for 3 children (10.7%) in the moxifloxacin group and 3 (9.7%) in the no intervention group.

### Description of Eligible Trials in the Systematic Review

**Figure 3.**
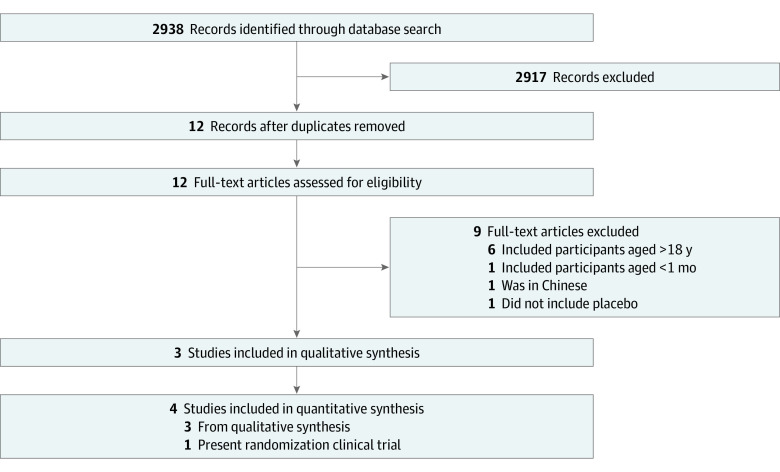
Flow Diagram of the Systematic Review and Meta-analysis

The database search revealed a total of 2938 records, 21 of which met the study criteria (Figure 3). Of these, 9 were excluded because they were duplicates, so that 12 full-text articles were left for eligibility assessment. A further 9 articles were then excluded after full assessment, 6 of which included adult patients; 1, patients younger than 1 month; 1, available only in Chinese; 1, not placebo-controlled. Overall, 3 previous studies^3,4,8^ and the present results were included in the analysis.

The total number of participants included in the meta-analysis was 584 (eTable 3 in Supplement 2). The trials performed by Gigliotti et al^3^ and Rose et al,^4^ together with the present study, were RCTs, whereas the work published by Comstock et al^8^ was a post hoc subgroup analysis of 3 RCTs, and only the data on children aged 1 to 5 years were included in the present meta-analysis. The trials by Gigliotti et al,^3^ Rose et al,^4^ and Comstock et al^8^ compared topical antibiotics with a topical placebo, whereas the present trial compared topical antibiotics with a topical placebo and with the absence of an intervention. The risk of bias was assessed to be low in the trials by Gigliotti et al,^3^ Rose et al,^4^ and the present RCT but moderate in the trial by Comstock et al^8^ (eTable 4 in Supplement 2). The analysis of the proportion of participants with conjunctival symptoms on days 3 to 6 had a heterogeneity of 25%. The funnel plot and Egger test showed no publication bias for the primary outcome (eFigure 1 in Supplement 2). Heterogeneities and funnel plots for secondary outcomes are reported in eFigures 2 to 5 in Supplement 2.

### Outcomes of the Meta-analysis

**Figure 4.**
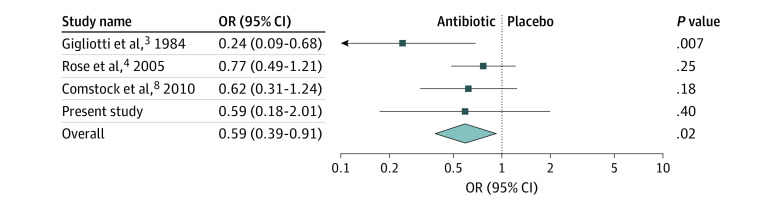
Proportions of Participants With Conjunctival Symptoms on Days 3 to 6 in Trials Comparing Antibiotics With a Placebo for Treating Acute Conjunctivitis in Children OR indicates odds ratio.

The proportion of children who were symptomatic on days 3 to 6 was reported in 4 studies, including the present RCT,^3,4,8^ and the meta-analysis showed that the use of topical antibiotics was associated with a significant reduction in the proportion of participants who had conjunctival symptoms on days 3 to 6 compared with those who had received the placebo (OR, 0.59; 95% CI, 0.39-0.91; *P* = .02) (Figure 4). The proportion of children who were symptomatic on days 7 to 10 was reported in 4 studies, including the present one,^3,4,8^ and the meta-analysis showed that the use of topical antibiotics was associated with a significant reduction in the proportion of children who had conjunctival symptoms on days 7 to 10 compared with those who had received the placebo (OR, 0.53; 95% CI, 0.34-0.83; *P* = .006) (eFigure 4 in Supplement 2).

Microbiological cures by days 7 to 10 were reported in 3 studies.^3,4,8^ The meta-analysis showed that the use of topical antibiotics was associated with a significant reduction in the proportion of participants who had a positive bacterial culture from the conjunctivae on days 7 to 10 compared with those who had received the placebo (OR, 0.34; 95% CI, 0.17-0.68; *P* = .003) (eFigure 5 in Supplement 2).

## Discussion

This systematic review and meta-analysis of combined data from the present trial and 3 previous studies^3,4,8^ found that the use of topical antibiotics was associated with a significant reduction in the proportion of children who had symptoms of conjunctivitis on days 3 to 6 compared with cases receiving a placebo. The evidence for this has previously been conflicting. Gigliotti et al^3^ showed in the early 1980s that treatment with polymyxin-bacitracin eye drops resulted in higher clinical cure rates on days 2 to 5 than treatment with a placebo, but when Rose and colleagues^4^ conducted a large trial consisting of 326 outpatient children with acute infective conjunctivitis in 2005, they found no significant difference in the cure rates at 1 week between the children treated with chloramphenicol eye drops and those treated with placebo eye drops. The present study, which was designed to address current inconsistencies, found that topical antibiotics were effective when used to treat acute infectious conjunctivitis in children.

An acknowledged weakness of previous RCTs^3,4^ has been that none of the trials have included a control group without an intervention. It has been suggested that placebo eye drops alone may be of benefit in the management of acute infective conjunctivitis because of their washout effect. Moreover, the largest trial of which we are aware found no difference in cure times between antibiotic eye drops and a placebo,^4^ but it included a placebo solution that contained preservatives with antiseptic properties. To avoid the bias caused by washout or anti-infective effects of a placebo, groups with no intervention at all and a placebo solution that did not contain any preservatives were included in the present RCT. As a result, the placebo eye drops significantly reduced the time to clinical cure in the present time-to-event analysis by comparison with no treatment, although the difference in the mean times to clinical cure between the groups was not statistically significant. These findings do suggest, however, that lubricating eye drops may make some contribution to the management of acute infective conjunctivitis in children.

The Cochrane collaboration conducted a systematic review and meta-analysis^5^ of the management of acute infective conjunctivitis in 2012 that led the authors to conclude that topical antibiotics can be of benefit for the treatment of conjunctivitis in that they improved the clinical cure rates both on days 2 to 5 and on days 6 to 10. That meta-analysis, however, included both adults and children, so that the results cannot be directly generalized to pediatric populations. To our knowledge, the present meta-analysis is the first to date to be based on data from pediatric patient populations alone.

Previous research into the etiology of acute infective conjunctivitis in children has found that approximately 80% of cases are caused by pathogenic bacteria.^1,4,9^ Accordingly, more than 80% of the children in this RCT had a positive bacterial conjunctival culture. Although most of the present children with acute infective conjunctivitis presented with respiratory tract symptoms, respiratory viruses were only detected in the conjunctivae of 12%, which is in accordance with previous observations.^1,4,9^

The strengths of this trial include a randomized, placebo-controlled study design with the inclusion of a control group receiving no treatment, which mimics a clinical situation. We then conducted a systematic review and meta-analysis of all RCTs we could find that were carried out with pediatric patient populations, including the RCT presented here. The results are directly applicable to primary care settings.

### Limitations

This study has limitations. We were not able to compare topical antibiotic therapy with no intervention in the meta-analysis because the present RCT was, to our knowledge, the only trial that had included a control group without an intervention. Moreover, the placebo solutions used in the 4 trials included in the meta-analysis varied, so that we could not assess possible differences between them. Furthermore, we could not compare the efficacy of topical antibiotic therapy separately in children with bacterial and viral conjunctivitis.

## Conclusions

In this RCT, use of topical antibiotics reduced the time to clinical cure, and in the meta-analysis of combined data from the present trial and previous RCTs, the use of topical antibiotics was associated with a significant reduction in the proportion of children who had symptoms of conjunctivitis on days 3 to 6 compared with those receiving a placebo. The present RCT results also suggest that lubricating eye drops may have some benefits for the management of acute infective conjunctivitis in children.

## Notes

Supplement 1.
Trial Protocol

Supplement 2. **eTable 1. **Baseline Characteristics of Participants in the Randomized Clinical Trial **eTable 2. **Microbiological Findings at Entry **eTable 3. **Descriptions of the Randomized Clinical Trials Included in the Systematic Review and Meta-analysis **eTable 4. **Risk of Bias Assessments in the Meta-analysis Material Using the Cochrane Collaboration’s Tool for Assessing the Risk of Bias in Randomized Trials **eFigure 1. **Funnel Plot of Studies Included for Assessment of the Proportion of Participants With Conjunctival Symptoms on Days 3 to 6 **eFigure 2. **Funnel Plot of Studies Included for Assessment of the Proportion of Participants With Conjunctival Symptoms on Days 7 to 10 **eFigure 3. **Funnel Plot of Studies Included for Assessment of the Proportion of Participants Who Had a Positive Bacterial Culture From the Conjunctivae on Days 7 to 10 **eFigure 4. **Proportions of Participants With Conjunctival Symptoms on Days 7 to 10 in Trials Comparing Antibiotics With a Placebo for Treating Acute Conjunctivitis in Children **eFigure 5. **Proportions of Participants Who Had a Positive Bacterial Culture From the Conjunctivae on Days 7 to 10 in Trials Comparing Antibiotics With a Placebo for Treating Acute Conjunctivitis in Children

Supplement 3.
Data Sharing Statement

## References

[1] Weiss A, Brinser JH, Nazar-Stewart V. Acute conjunctivitis in childhood. J Pediatr. 1993;122(1):10-14. doi:10.1016/S0022-3476(05)83479-1 8419593

[2] Rietveld RP, ter Riet G, Bindels PJ, Schellevis FG, van Weert HC. Do general practitioners adhere to the guideline on infectious conjunctivitis? results of the Second Dutch National Survey of General Practice. BMC Fam Pract. 2007;8:54. doi:10.1186/1471-2296-8-54 17868475 PMC2034564

[3] Gigliotti F, Hendley JO, Morgan J, Michaels R, Dickens M, Lohr J. Efficacy of topical antibiotic therapy in acute conjunctivitis in children. J Pediatr. 1984;104(4):623-626. doi:10.1016/S0022-3476(84)80566-1 6323667

[4] Rose PW, Harnden A, Brueggemann AB, . Chloramphenicol treatment for acute infective conjunctivitis in children in primary care: a randomised double-blind placebo-controlled trial. Lancet. 2005;366(9479):37-43. doi:10.1016/S0140-6736(05)66709-8 15993231

[5] Sheikh A, Hurwitz B, van Schayck CP, McLean S, Nurmatov U. Antibiotics versus placebo for acute bacterial conjunctivitis. Cochrane Database Syst Rev. 2012;9(9):CD001211. doi:10.1002/14651858.CD001211.pub3 22972049

[6] Moher D, Liberati A, Tetzlaff J, Altman DG; PRISMA Group. Preferred reporting items for systematic reviews and meta-analyses: the PRISMA statement. PLoS Med. 2009;6(7):e1000097. doi:10.1371/journal.pmed.1000097 19621072 PMC2707599

[7] Higgins JP, Altman DG, Gøtzsche PC, ; Cochrane Bias Methods Group; Cochrane Statistical Methods Group. The Cochrane Collaboration’s tool for assessing risk of bias in randomised trials. BMJ. 2011;343:d5928. doi:10.1136/bmj.d5928 22008217 PMC3196245

[8] Comstock TL, Paterno MR, Usner DW, Pichichero ME. Efficacy and safety of besifloxacin ophthalmic suspension 0.6% in children and adolescents with bacterial conjunctivitis: a post hoc, subgroup analysis of three randomized, double-masked, parallel-group, multicenter clinical trials. Paediatr Drugs. 2010;12(2):105-112. doi:10.2165/11534380-000000000-00000 20218747

[9] Gigliotti F, Williams WT, Hayden FG, . Etiology of acute conjunctivitis in children. J Pediatr. 1981;98(4):531-536. doi:10.1016/S0022-3476(81)80754-8 6970802

