# Supplementary material for: Effect of Topical Antibiotics on Duration of Acute Infective Conjunctivitis in Children: A Randomized Clinical Trial and a Systematic Review and Meta-analysis
Source: JAMA Netw Open. 2022 Oct 4;5(10):e2234459. doi: 10.1001/jamanetworkopen.2022.34459 (PMC9533187; doi:10.1001/jamanetworkopen.2022.34459)
Supplement: Supplement 1. — Trial Protocol [file jamanetwopen-e2234459-s001.pdf]

Topical Antibiotics for Acute Infective Conjunctivitis in Children: A Randomized Clinical Trial and a Systematic Review and Meta-analysis

Study Protocol and Statistical Analysis Plan

Minna Honkila[1,2], M.D., Ph.D., Ulla Koskela[1,2], M.D., Ph.D., Tero Kontiokari[3], M.D., Ph.D., Marja-Leena Mattila[3], M.D., Ph.D., Aila Kristo[3], M.D., Ph.D., Raija Valtonen[4], M.D., Suvi Sarlin[1,2], M.D., Tytti Pokka[1,2], M.Sc., Niko Paalanne[1,2], M.D., Ph.D., Irma Ikäheimo[5], Ph.D., Matti Uhari[1,2], M.D., Ph.D., Marjo Renko[6], M.D., Ph.D., Terhi Tapiainen[1,2,7], M.D., Ph.D.

1. Department of Pediatrics and Adolescent Medicine, Oulu University Hospital, Oulu, Finland
2. Research Unit of Clinical Medicine and Medical Research Centre Oulu, University of Oulu, Oulu, Finland
3. Mehiläinen Oulu, Oulu, Finland
4. Terveystalo Oulu, Oulu, Finland
5. NordLab Oulu, Oulu, Finland
6. University of Eastern Finland and Kuopio University Hospital, Kuopio, Finland
7. Biocenter Oulu, University of Oulu, Oulu, Finland

Contact information:

Minna Honkila, M.D., Ph.D., pediatric infectious diseases specialist
Department of Pediatrics and Adolescent Medicine
Oulu University Hospital
P.O. Box 23
FIN-90029 Oulu University Hospital, Finland
email: minna.honkila@oulu.fi, telephone: +358-9-3155863

1. INTRODUCTION

1.1. Background

Acute conjunctivitis is a common childhood infection which is usually viral or bacterial in etiology. According to previous studies, around 80% of cases are caused by bacteria and 13-20% by viruses.[1-2] Bacterial conjunctivitis is commonly due to *Haemophilus influenzae*, *Streptococcus pneumoniae*, *Moraxella catarrhalis*, or *Staphylococcus aureus*, whereas viral conjunctivitis is usually caused by adenoviruses.[1-4] It is, however, difficult to differentiate on clinical grounds whether the infection is viral or bacterial.

Topical antibiotics are a standard clinical practice for the treatment of acute infective conjunctivitis,[5] because they seem to speed recovery and reduce relapse.[6] However, the benefits of antibiotic therapy in the management of childhood conjunctivitis are poorly documented. There are only two previous randomized controlled trials that have investigated topical antimicrobial therapy in acute infective conjunctivitis in children. Gigliotti et al. reported in their study comprising 102 children aged 1 month to 18 years from the late 1970's and early 1980's that treatment with polymyxin-bacitracin resulted in greater clinical and microbiological remission at days 2 to 5 compared with placebo treatment.[4] On the other hand, Rose et al. found in their trial consisting of 326 children that there was no difference in the clinical cure rate by day 7 between children receiving a placebo and those receiving chloramphenicol.[1]

1.2. Objectives

The aims of this study are to investigate the efficacy of topical antibiotic therapy compared with either placebo eye drops or no treatment for the management of acute conjunctivitis in children and to assess the microbiological etiology of acute conjunctivitis.

2. MATERIAL AND METHODS

2.1. Trial design

The study is a single center randomized controlled trial comparing topical antibiotic therapy with a topical placebo and with no intervention.

2.2. Participants and setting

The trial will include children 6 months to 7 years of age with a clinical diagnosis of acute conjunctivitis (defined as the presence of conjunctival discharge, erythema, soreness or swelling of the eyelids). The exclusion criteria are allergy to fluoroquinolones, antibiotic therapy 7 days prior to the trial, severe infection, allergic conjunctivitis, or a trauma or a foreign body in the eye. The study will be conducted at 2 pediatric outpatient clinics in the city of Oulu and at the pediatric emergency department of Oulu University Hospital, Oulu, Finland.

2.3. Interventions

The children deemed eligible will be randomly assigned in a 1:1:1 ratio to receive moxifloxacin eye drops (Vigamox 5 mg/mL; Novartis Finland, Espoo, Finland), placebo eye drops (Celluvisc 1.0%; Allergan Pharmaceuticals Ireland, Westport, Co. Mayo, Ireland), or no intervention. Both moxifloxacin and placebo eye drops are to be applied one drop 3 times daily until the symptoms have been absent for at least 24 hours but no longer than 7 days. All participants will be advised to remove discharge from the eyes at least 3 times a day and before applying the eye drops.

2.4. Blinding

The children participating in the trial, their parents and the physicians and nurses involved will be blinded to the medications applied but not to the choice of no treatment because of the obvious nature of the intervention. Moxifloxacin eye drops are packed in 5 mL plastic dropper bottles with a screw top and the placebo eye drops in 0.4 mL single-dose plastic vials with flip off seals. Both the bottles and the vials are transparent and do not have any labels on them. The eye drop bottles and vials will then be packed into opaque cardboard boxes which will also contain instructions for applying the eye drops.

2.5. Procedures

The children will be recruited by pediatricians working in private health care clinics in Oulu, Finland (TK, M-LM, AK, RV), and conjunctival and nasopharyngeal specimens will be obtained from them by the laboratory personnel at those clinics. An instruction sheet about sampling will be prepared and distributed to the laboratory personnel. In addition, all the materials needed for specimen collection will be packed in ready-for-use sets. Parents will be asked to complete a questionnaire about their child’s current condition and to continue the follow-up for 14 days using the daily symptom sheet diary. In addition, the research physician (MH) will send an SMS to parents after 14 days of follow-up to verify the absence of symptoms. Parents can contact this physician any time during the trial if they are concerned about the child´s condition. Moxifloxacin eye drops will be started in a non-blinded manner as a rescue treatment if needed.

Transystem M40 transport cotton tipped swabs will be used for bacterial culture and flocked swabs (Copan FLOQSwabs; Copan Diagnostics, Inc) for respiratory pathogen polymerase chain reaction (PCR) testing. Conjunctival specimens will be collected from the actual site of infection, and from both eyes in cases of bilateral conjunctivitis. The area around the affected eye will be cleansed to remove discharge, after which the culture material will be obtained by swabbing the mucosal area of the lower

eyelid. After that, the inner surface of the lower eyelid is to be swabbed thoroughly two to three times to collect epithelial cells for nucleic acid amplification testing. Nasopharyngeal swabs will be obtained from each child by passing the swab through the nostril to the nasopharynx and rotating it at least two to three times to collect epithelial cells. The swab for respiratory viruses will be inserted into a 3 mL transport medium tube (Universal Transport Media, UTM™; Copan Diagnostics, Inc). The swabs will be stored and transported at room temperature to the clinical microbiological laboratory at Oulu University Hospital (NordLab, Oulu) on the same working day.

Sheep blood agars at a concentration of 5% and chocolate agars will be used throughout to culture the bacteria. In addition, mass spectrometry (VITEK, bioMerieux) will be used to identify the bacteria from 2018 onwards.

A multiplex real-time PCR will be used to detect respiratory viruses. From 15 October 2014 to 31 December 2019 the panel will include adenovirus, bocavirus, enteroviruses, influenza viruses A and B, corona viruses 229E, Nl63, and OC43, human metapneumovirus, parainfluenza viruses 1, 2, 3 and 4, respiratory syncytial viruses A and B, and rhinovirus, while from 1 January 2020 onwards it will include influenza A, influenza A subtypes H1N1, H1 and H3, influenza B, corona viruses 229E, HKU1, NL63 and OC43, parainfluenza viruses 1, 2, 3 and 4, respiratory syncytial virus, human metapneumovirus, adenovirus, bocavirus, rhinovirus/enterovirus, *Mycoplasma pneumoniae*, *Legionella pneumophila* and *Bordetella pertussis*. Nucleic acid isolation for respiratory pathogens will be performed using the QIAsymphony DSP Virus/Pathogen Mini Kit (Qiagen) and a QIAsymphony SP instrument (Qiagen). The nucleic acid amplification and detection will take place using AnyplexTM II RV16 Detection (Seegene, Inc) and the CF96TM Real-Time PCR System (Bio-Rad Laboratories) from 15 October 2014 to 31 December 2019. Between 9 January 2020 and 7 February 2020, a QIAstat-

DxTM (DiagCORE®) Respiratory Panel V2 (Qiagen) and the CF96TM Real-Time PCR System (Bio-Rad laboratories, Inc.) will be used for detection.

Amendment on 2 October 2017: We will prepare handouts with a brief outline of the research and distribute them to 49 day-care centers located in the city of Oulu, thereby contacting more than 4000 families. If interested, a family can contact the research team directly by telephone. The team at Oulu University Hospital (MH, UK, NP, SS) will recruit eligible children for the trial and nurses working in the pediatric emergency room at Oulu University Hospital will obtain conjunctival and nasopharyngeal specimens from these children.

2.6. Outcomes

The primary outcome is time (in days) to a clinical cure, defined as resolution of all the conjunctival symptoms for 2 days without relapse. The secondary outcome is a relapse of conjunctivitis within 14 days of randomization. We also will record any discomfort caused by administration of the eye drops and the effect of conjunctivitis on the quality of life (employing a visual analogue scale).

3. STATISTICAL ANALYSIS PLAN

3.1. Sample size calculation

During the registration process at ClinicalTrialsRegister.eu we evaluated the sample size required to obtain a 1-day difference in cure times between the groups. After the registration process but prior to initiation of the trial, we estimated that acute infective conjunctivitis in children resolves itself in 5 days (SD 2) without treatment,[1] so that a 1.5-day difference in cure time can be considered clinically significant. With a statistical power of 80% and a 2-sided α error of 0.05, 29 children per group would be needed for the trial.

All the analyses will be performed on an intention-to-treat population, and only the primary and secondary outcomes that are prespecified in the protocol and statistical analysis plan will be compared. A one-way ANOVA test will be used to compare the duration of symptoms and Kaplan-Meier survival statistics to assess the variation in cure times. Relapse rates in the three groups will be compared using the Chi-square test. The data will be analyzed with IBM SPSS Statistics 25.

4. ORGANIZATION, FUNDING AND SCHEDULE

The leaders of this independent investigator-initiated trial, Dr Minna Honkila, M.D., Ph.D., and Dr Terhi Tapiainen, M.D., Ph.D., are specialists in pediatric infectious diseases working at Oulu University Hospital and the University of Oulu. All the recruiting doctors are experienced clinicians, and the research team is experienced in conducting clinical trials among pediatric patients. Funding has been applied for from various foundations to cover the costs of medications involved and to enable full-time research for several months (Dr Minna Honkila). The work will begin in spring 2014.

Amendment on 7 February 2020: Due to the COVID-19 pandemic in spring 2020 and the national recommendations to restrict visits to healthcare facilities, we have decided to end the trial in March 2020.

Amendment on 19 June 2021: **Meta-analysis**

We will identify the literature in PubMed, ClinicalTrials.gov, Cochrane Library, Google Scholar, Scopus and ScienceDirect from inception to 31 December 2020 using the MeSH (Medical Subjects Headings) terms "conjunctivitis, bacterial" not "trachoma" for a literature search and include all randomized controlled trials that have compared topical antibiotics with a control group without

antibiotics for the treatment of acute conjunctivitis in children and adolescents aged 1 month to 18 years. We will use PRISMA (PRISMA 2009 Checklist and PRISMA 2009 Flow Diagram) as a basis for reporting the meta-analysis and aim to collect, check, and reanalyze individual participation data for each eligible subject.

Our primary outcome is the time (days) to a clinical cure. Secondary outcomes are time (days) to a microbiological cure, treatment compliance, relapse of conjunctivitis within 4 weeks, complications and treatment of acute otitis and reports of adverse events.

Amendment on 9 November 2021: As it is not possible to obtain data on the original primary and secondary outcomes from the articles included in the meta-analysis, we modified the outcomes as follows: the primary outcome of the meta-analysis is the proportion of participants who has conjunctival symptoms on days 3 to 6, while secondary outcomes are the proportion of participants who has conjunctival symptoms on days 7 to 10 and the proportion without a microbiological cure on days 7 to 10.

Data from randomized controlled trials will be used to calculate odds ratios and 95% confidence intervals comparing antibiotics with a placebo and with no treatment. The χ^2 heterogeneity test and I^2 statistic will be used to investigate heterogeneity between trials. Potential publication bias will be analyzed using funnel plots and the Egger test. The analyses of outcomes will be performed using Comprehensive Meta Analysis software version 3.3.070 (Biostat, Inc).

References

1. Rose PW, Harnden A, Brueggemann AB, et al. Chloramphenicol treatment for acute infective conjunctivitis in children in primary care: a randomised double-blind placebo-controlled trial. *Lancet*. 2005;366(9479):37-43.
2. Gigliotti F, Williams WT, Hayden FG, et al. Etiology of acute conjunctivitis in children. *J Pediatr*. 1981;98(4):531-536.
3. Hautala N, Koskela M, Hautala T. Major age group-specific differences in conjunctival bacteria and evolution of antimicrobial resistance revealed by laboratory data surveillance. *Curr Eye Res*. 2008;33(11):907-911.
4. Gigliotti F, Hendley JO, Morgan J, Michaels R, Dickens M, Lohr J. Efficacy of topical antibiotic therapy in acute conjunctivitis in children. *J Pediatr*. 1984;104(4):623-626.
5. Rietveld RP, ter Riet G, Bindels PJ, Schellevis FG, van Weert HC. Do general practitioners adhere to the guideline on infectious conjunctivitis? Results of the Second Dutch National Survey of General Practice. *BMC Fam Pract*. 2007;8:54.
6. Sheikh A, Hurwitz B, van Schayck CP, McLean S, Nurmatov U. Antibiotics versus placebo for acute bacterial conjunctivitis. *Cochrane Database of Systematic Reviews*. 2012;9:CD001211.

LIST OF AMENDMENTS

Amendment on 2 October 2017:

We will prepare handouts with a brief outline of the research and distribute them to 49 day-care centers located in the city of Oulu, thereby contacting more than 4000 families. If interested, a family can contact the research team directly by telephone. The team at Oulu University Hospital (MH, UK, NP, SS) will recruit eligible children for the trial and nurses working in the pediatric emergency room at Oulu University Hospital will obtain conjunctival and nasopharyngeal specimens from these children.

Amendment on 7 February 2020:

Due to the COVID-19 pandemic in spring 2020 and the national recommendations to restrict visits to healthcare facilities, we have decided to end the trial in March 2020. In addition, we have decided to perform a meta-analysis of the treatment of acute conjunctivitis in children.

Amendment on 19 June 2021:

We will identify the literature in PubMed, ClinicalTrials.gov, Cochrane Library, Google Scholar, Scopus and ScienceDirect from inception to 31 December 2020 using the MeSH (Medical Subjects Headings) terms “conjunctivitis, bacterial” not “trachoma” for a literature search and include all randomized controlled trials that have compared topical antibiotics with a control group without antibiotics for the treatment of acute conjunctivitis in children and adolescents aged 1 month to 18 years. We will use PRISMA (PRISMA 2009 Checklist and PRISMA 2009 Flow Diagram) as a basis for reporting the meta-analysis and aim to collect, check, and reanalyze individual participation data for each eligible subject.

Our primary outcome is the time (days) to a clinical cure. Secondary outcomes are time (days) to a microbiological cure, treatment compliance, relapse of conjunctivitis within 4 weeks, complications of acute otitis and reports of adverse events.

Data from randomized controlled trials will be used to calculate odds ratios and 95% confidence intervals comparing antibiotics with a placebo and with no treatment. The χ^2 heterogeneity test and I^2 statistic will be used to investigate heterogeneity between trials. Potential publication bias will be analyzed using funnel plots and the Egger test. The analyses of outcomes will be performed using Comprehensive Meta Analysis software version 3.3.070 (Biostat, Inc).

Amendment on 9 November 2021:

As it is not possible to obtain data on the original primary and secondary outcomes from the articles included in the meta-analysis, we modified the outcomes as follows: the primary outcome of the meta-analysis is the proportion of participants who has conjunctival symptoms on days 3 to 6, while secondary outcomes are the proportion of participants who has conjunctival symptoms on days 7 to 10 and the proportion without a microbiological cure on days 7 to 10.
