# Supplementary material for: Effect of Topical Antibiotics on Duration of Acute Infective Conjunctivitis in Children: A Randomized Clinical Trial and a Systematic Review and Meta-analysis
Source: JAMA Netw Open. 2022 Oct 4;5(10):e2234459. doi: 10.1001/jamanetworkopen.2022.34459 (PMC9533187; doi:10.1001/jamanetworkopen.2022.34459)
Supplement: Supplement 2. — eTable 1. Baseline Characteristics of Participants in the Randomized Clinical Trial eTable 2. Microbiological Findings at Entry eTable 3. Descriptions of the Randomized Clinical Trials Included in the Systematic Review and Meta-analysis eTable 4. Risk of Bias Assessments in the Meta-analysis Material Using the Cochrane Collaboration’s Tool for Assessing the Risk of Bias in Randomized Trials eFigure 1. Funnel Plot of Studies Included for Assessment of the Proportion of Participants With Conjunctival Symptoms on Days 3 to 6 eFigure 2. Funnel Plot of Studies Included for Assessment of the Proportion of Participants With Conjunctival Symptoms on Days 7 to 10 eFigure 3. Funnel Plot of Studies Included for Assessment of the Proportion of Participants Who Had a Positive Bacterial Culture From the Conjunctivae on Days 7 to 10 eFigure 4. Proportions of Participants With Conjunctival Symptoms on Days 7 to 10 in Trials Comparing Antibiotics With a Placebo for Treating Acute Conjunctivitis in Children eFigure 5. Proportions of Participants Who Had a Positive Bacterial Culture From the Conjunctivae on Days 7 to 10 in Trials Comparing Antibiotics With a Placebo for Treating Acute Conjunctivitis in Children [file jamanetwopen-e2234459-s002.pdf]

Supplemental Online Content

Honkila M, Koskela U, Kontiokari T, et al. Effect of topical antibiotics on duration of acute infective conjunctivitis in children: a randomized clinical trial and a systematic review and meta-analysis. *JAMA Netw Open.* 2022;5(10):e2234459. doi:10.1001/jamanetworkopen.2022.34459

eTable 1. Baseline Characteristics of Participants in the Randomized Clinical Trial

eTable 2. Microbiological Findings at Entry

eTable 3. Descriptions of the Randomized Clinical Trials Included in the Systematic Review and Meta-analysis

eTable 4. Risk of Bias Assessments in the Meta-analysis Material Using the Cochrane Collaboration's Tool for Assessing the Risk of Bias in Randomized Trials

eFigure 1. Funnel Plot of Studies Included for Assessment of the Proportion of Participants With Conjunctival Symptoms on Days 3 to 6

eFigure 2. Funnel Plot of Studies Included for Assessment of the Proportion of Participants With Conjunctival Symptoms on Days 7 to 10

eFigure 3. Funnel Plot of Studies Included for Assessment of the Proportion of Participants Who Had a Positive Bacterial Culture From the Conjunctivae on Days 7 to 10

eFigure 4. Proportions of Participants With Conjunctival Symptoms on Days 7 to 10 in Trials Comparing Antibiotics With a Placebo for Treating Acute Conjunctivitis in Children

eFigure 5. Proportions of Participants Who Had a Positive Bacterial Culture From the Conjunctivae on Days 7 to 10 in Trials Comparing Antibiotics With a Placebo for Treating Acute Conjunctivitis in Children

This supplemental material has been provided by the authors to give readers additional information about their work.

© 2022 Honkila M et al. *JAMA Network Open.*

eTable 1. Baseline Characteristics of Participants in the Randomized Clinical Trial

	**Moxiloxacin n = 30**	**Placebo n = 27**	**No intervention n = 31**
Age, mean (SD), years	2.8 (1.6)	3.0 (1.3)	3.2 (1.8)
Sex, n (%)			
Girls	17 (57)	13 (48)	16 (52)
Boys	13 (43)	14 (52)	15 (48)
Mother's education, n	**n = 29**	**n = 26**	**n = 31**
Basic education	0	1	1
Vocational education	4	7	6
General upper secondary education	0	0	1
Higher education	25	18	23
Father's education, n	**n = 28**	**n = 24**	**n = 31**
Basic education	2	0	0
Vocational education	6	10	14
General upper secondary education	0	1	0
Higher education	20	13	17
No. of siblings, mean (SD)	0.97 (1.09)	1.04 (0.96)	0.71 (0.59)
**Underlying medical condition, n (%)**			
Asthma	0 (0)	1 (3.7)	1 (3.2)
Atopic eczema	1 (3.3)	0 (0)	1 (3.2)
Suspected tear duct problem	0 (0)	0 (0)	2 (6.5)
Food allergy	1 (3.3)	1 (3.7)	0 (0)
Allergic rhinitis	1 (3.3)	0 (0)	0 (0)
Previous conjunctivitis, n (%)	13 (43)	16 (59)	19 (61)
Conjunctival erythema	29 (97)	24 (89)	30 (97)
Conjunctival discharge	25 (83)	20 (74)	26 (84)
Swelling of the eyelids	11 (37)	7 (26)	16 (52)
Bilateral conjunctivitis	25 (83)	20 (74)	24 (77)
Respiratory tract infection	1 (3.3)	5 (19)	4 (13)
Otitis media[a]	10 (33)	9 (33)	5 (16)

© 2022 Honkila M et al. *JAMA Network Open.*

	Moxiloxacin<br>n = 30	Placebo<br>n = 27	No intervention<br>n = 31
**Symptoms reported by guardians, n (%)**			
Conjunctival erythema	25 (83)	24 (89)	29 (94)
Conjunctival discharge	27 (90)	23 (85)	27 (87)
Swelling of the eyelids	12 (40)	10 (37)	18 (58)
Soreness	17 (57)	16 (59)	16 (52)
**Other symptoms, n (%)**			
Rhinorrhea	24 (80)	19 (70)	22 (71)
Cough	18 (60)	19 (70)	18 (58)
Earache	5 (17)	6 (22)	4 (13)
Fever > 38	2 (6.7)	3 (11)	7 (23)
Irritability	0 (0)	2 (7.4)	0 (0)
Headache	1 (3.3)	0 (0)	0 (0)
Sore throat	0 (0)	0 (0)	1 (3.2)
Stuffy nose	0 (0)	0 (0)	1 (3.2)
Vomiting and diarrhea	0 (0)	0 (0)	1 (3.2)

[a]All were treated with systemic antibiotics (most with amoxicillin: 9/10 in the moxifloxacin group, 9/10 in the placebo group, and 3/5 in the no intervention group).

© 2022 Honkila M et al. *JAMA Network Open.*

eTable 2. Microbiological Findings at Entry

	Moxifloxacin[a]	Placebo[b]	No intervention[c]	Total
**Viruses**				
Nasopharyngeal sample available	**n = 28**	**n = 25**	**n = 31**	**N = 84**
At least one virus detected, n (%)	16 (57)	17 (68)	13 (42)	46 (55)
Rhinovirus	9 (32)	7 (28)	6 (19)	22 (26)
Bocavirus	6 (21)	2 (8.0)	1 (3.2)	9 (11)
Adenovirus	1 (3.6)	3 (12)	4 (13)	8 (9.5)
Coronavirus HKU1/NL63/OC43	3 (11)	3 (12)	0 (0)	6 (7.1)
Parainfluenzavirus types 3, 4	0 (0)	1 (4.0)	3 (9.7)	4 (4.7)
Enterovirus	0 (0)	1 (4.0)	1 (3.2)	2 (2.4)
Rhinovirus/enterovirus	0 (0)	2 (8.0)	0 (0)	2 (2.4)
Human metapneumovirus	0 (0)	1 (4.0)	0 (0)	1 (1.2)
RSV	0 (0)	1 (4.0)	0 (0)	1 (1.2)
Conjunctival sample available	**n = 28**	**n = 25**	**n = 31**	**N = 84**
At least one virus detected, n (%)	5 (18)	3 (12)	2 (6.5)	10 (12)
Bocavirus	3 (11)	1 (4.0)	0 (0)	4 (4.8)
Rhinovirus/enterovirus	2 (7.1)	0 (0)	2 (6.5)	4 (4.8)
Coronavirus HKU1/OC43	1 (3.6)	1 (4.0)	0 (0)	2 (2.4)
Adenovirus	0 (0)	1 (4.0)	0 (0)	1 (1.2)
Human metapneumovirus	1 (3.6)	0 (0)	0 (0)	1 (1.2)
**Bacteria**				
Conjunctival sample available	**n = 29**	**n = 25**	**n = 30**	**N = 84**
At least one pathogen detected, n (%)	27 (93)	19 (76)	24 (80)	70 (83)
Haemophilus influenzae	24 (83)	15 (60)	19 (63)	58 (69)
Streptococcus pneumoniae	4 (14)	2 (8.0)	4 (13)	10 (12)
Staphylococcus aureus	0 (0)	5 (20)	3 (10)	8 (9.5)
Moraxella catarrhalis	0 (0)	1 (4.0)	1 (3.3)	2 (2.4)
Group A streptococcus	0 (0)	0 (0)	1 (3.3)	1 (1.2)
Group C streptococcus	0 (0)	1 (4.0)	0 (0)	1 (1.2)

[a]1 child had 2 bacteria in the conjunctivae, 2 children had 2 viruses in the conjunctivae and 3 children had 2 viruses in the nasopharynx.
[b]5 children had 2 bacteria in the conjunctivae, 3 children had 2 viruses in the nasopharynx and 1 child had 3 viruses in the nasopharynx.
[c]3 children had 2 bacteria in the conjunctivae and 2 children had 2 viruses in the nasopharynx.

© 2022 Honkila M et al. *JAMA Network Open.*

eTable 3. Descriptions of the Randomized Clinical Trials Included in the Systematic Review and Meta-analysis

**Author, year**	**Gigliotti et al,[3] 1984**	**Rose et al,[4] 2005**	**Comstock et al,[8] 2010**	**Present study**
Antimicrobial agent	Polymyxin-bacitracin	Chloramphenicol 0.5%	Besifloxacin 0.6% (includes benzalkonium chloride 0.01%)	Moxifloxacin
Placebo	Vehicle without antibiotics	Distilled water with the excipients boric acid (1.5%) and borax (0.3%)	Vehicle without antibiotics	Artificial tears containing carmellose sodium, sodium chloride, sodium lactate, potassium chloride and purified water
No. of patients in antibiotic group	34	163	73	30
No. of patients in placebo group	32	163	62	27
No. of patients in no treatment group	NR	NR	NR	31
Mean (SD) age of antibiotic group	NR	3.3 (2.8)	NR (range 1-5 y)	2.8 (1.6)
Mean (SD) age of placebo group	NR	3.3 (2.6)	NR (range 1-5 y)	3.0 (1.3)
Mean (SD) age of no treatment group	NR	NR	NR	3.2 (1.8)
Clinically cured on days 3-6 in antibiotic group	21/34	64/163	45/73	24/30
Clinically cured on days 3-6 in placebo group	9/32	54/163	31/62	19/27
Clinically cured on days 3-6 in no treatment group	NR	NR	NR	15/31
Clinically cured on days 7-10 in antibiotic group	31/34	140/163	64/73	28/30
Clinically cured on days 7-10 in placebo group	23/32	128/163	47/62	26/27

© 2022 Honkila M et al. *JAMA Network Open.*

Author, year	Gigliotti et al,[3] 1984	Rose et al,[4] 2005	Comstock et al,[8] 2010	Present study
Clinically cured on days 7-10 in no treatment group	NR	NR	NR	27/31
Duration of eye symptoms in antibiotic group	NR	5.0 (1.9)	NR	3.8 (3.1)
Duration of eye symptoms in placebo group	NR	5.4 (1.9)	NR	4.0 (2.3)
Duration of eye symptoms in no treatment group	NR	NR	NR	5.7 (3.3)
Microbiologically cured on days 7-10 in antibiotic group	27/34	50/125	55/73	NR
Microbiologically cured on days 7-10 in placebo group	10/32	29/125	37/62	NR
Microbiologically cured on days 7-10 in no treatment group	NR	NR	NR	NR
Bacterial pathogens in the conjunctivae	61 *Haemophilus influenzae*<br>22 *Streptococcus pneumoniae*<br>1 both	197 *Haemophilus influenzae*<br>64 *Streptococcus pneumoniae*<br>36 *Moraxella catarrhalis*	NR	58 *Haemophilus influenzae*<br>10 *Streptococcus pneumoniae*<br>8 *Staphylococcus aureus*<br>2 *Moraxella catarrhalis*<br>1 Group A streptococcus<br>1 Group C streptococcus
Viral pathogens in the conjunctivae		19 adenovirus<br>24 picornavirus	NR	4 bocavirus<br>3 rhinovirus<br>2 coronavirus (not SARS-CoV-2)<br>1 adenovirus<br>1 enterovirus<br>1 human metapneumovirus

© 2022 Honkila M et al. *JAMA Network Open*.

Abbreviations: NR, not reported.

© 2022 Honkila M et al. *JAMA Network Open*.

eTable 4. Risk of Bias Assessments in the Meta-analysis Material Using the Cochrane Collaboration's Tool for Assessing the Risk of Bias in Randomized Trials[a]

	Random sequence generation	Allocation concealment	Blinding of participants and personnel	Blinding of outcome assessment	Incomplete outcome data	Selective reporting	Other bias
Gigliotti et al,[3] 1984	?	+	+	+	?	–	+
Rose et al,[4] 2005	+	+	+	+	+	+	–
Comstock et al,[8] 2010	+	?	?	–	–	–	–
Present study[b]	+	+	+	+	+	+	+

Abbreviations: +, low risk of bias; –, high risk of bias; ?, unclear risk of bias.
[a]Higgins JP, Altman DG, Gøtzsche PC, et al; Cochrane Bias Methods Group; Cochrane Statistical Methods Group. The Cochrane Collaboration's tool for assessing risk of bias in randomised trials. *BMJ*. 2011;343:d5928.
[b]Self-assessment.

© 2022 Honkila M et al. *JAMA Network Open.*

eFigure 1. Funnel Plot of Studies Included for Assessment of the Proportion of Participants With Conjunctival Symptoms on Days 3 to 6

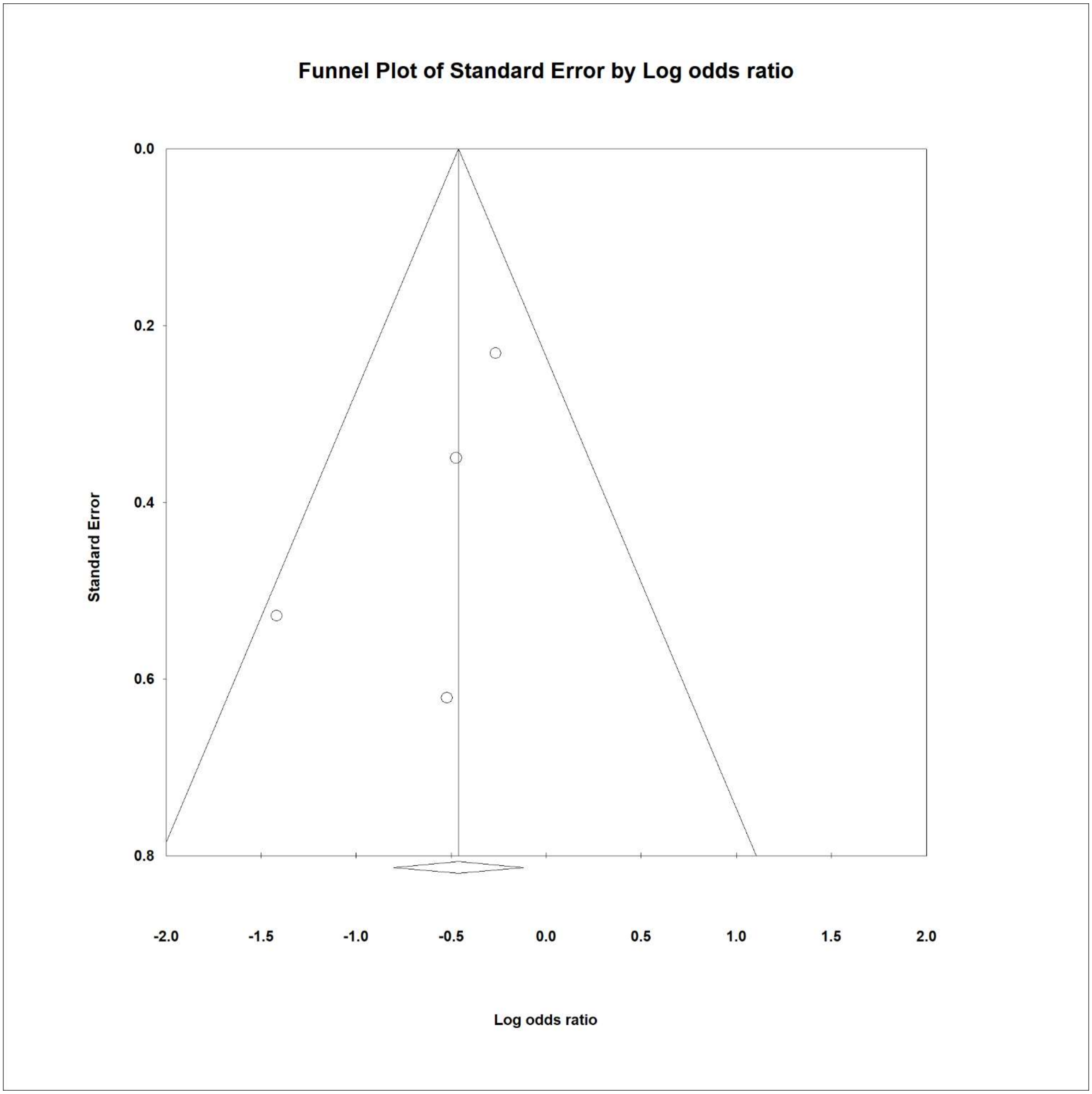

Egger test: P = 0.26

© 2022 Honkila M et al. *JAMA Network Open.*

eFigure 2. Funnel Plot of Studies Included for Assessment of the Proportion of Participants With Conjunctival Symptoms on Days 7 to 10

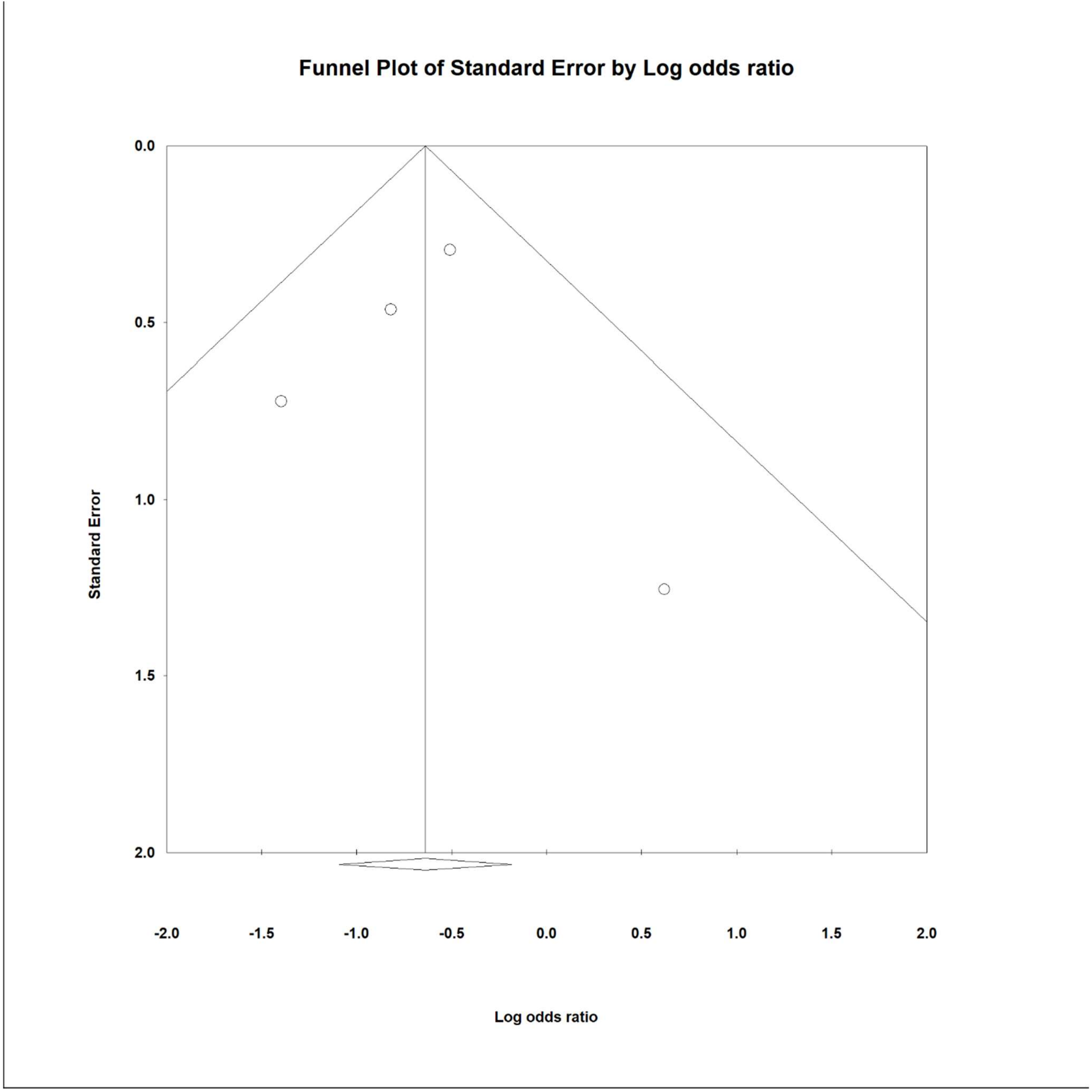

Egger test: P = 1.00

© 2022 Honkila M et al. *JAMA Network Open.*

eFigure 3. Funnel Plot of Studies Included for Assessment of the Proportion of Participants Who Had a Positive Bacterial Culture From the Conjunctivae on Days 7 to 10

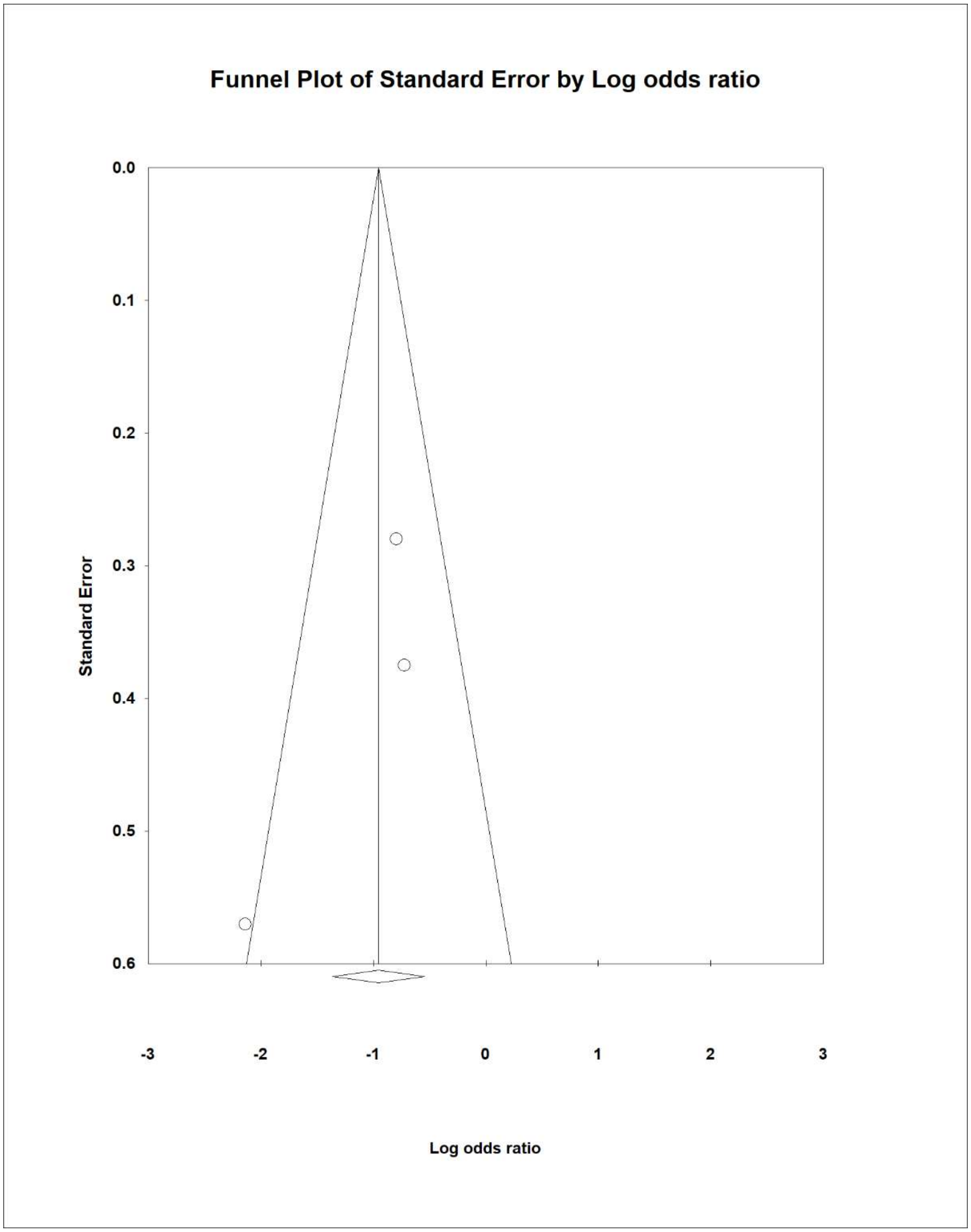

Harbord-Egger test: P = 0.39

© 2022 Honkila M et al. *JAMA Network Open.*

eFigure 4. Proportions of Participants With Conjunctival Symptoms on Days 7 to 10 in Trials Comparing Antibiotics With a Placebo for Treating Acute Conjunctivitis in Children

Study name	Odds ratio	Lower limit	Upper limit	p-Value
Gigliotti et al,[3] 1984	0.25	0.06	1.02	0.053
Rose et al,[4] 2005	0.60	0.34	1.07	0.084
Comstock et al,[8] 2010	0.44	0.18	1.09	0.077
Present study	1.86	0.16	21.72	0.622
	0.53	0.34	0.83	0.006

Odds ratio and 95% CI

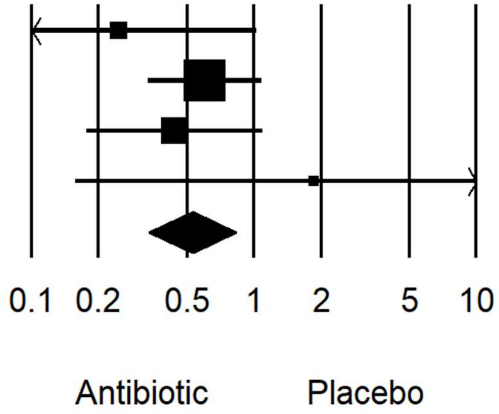

© 2022 Honkila M et al. *JAMA Network Open.*

eFigure 5. Proportions of Participants Who Had a Positive Bacterial Culture From the Conjunctivae on Days 7 to 10 in Trials Comparing Antibiotics With a Placebo for Treating Acute Conjunctivitis in Children

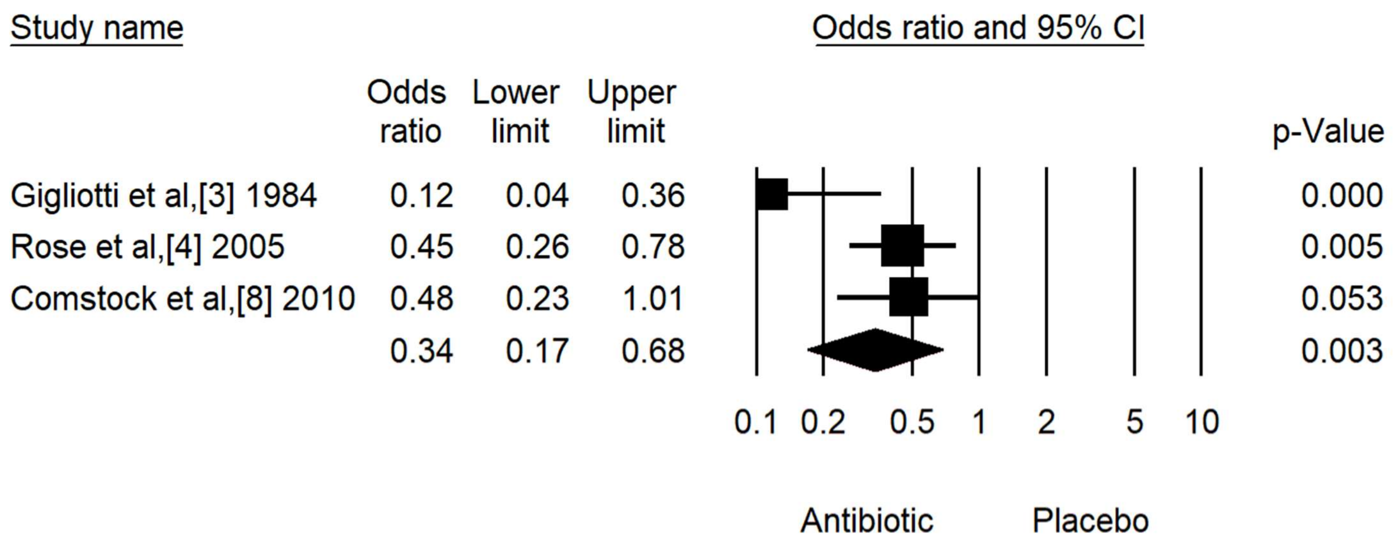

© 2022 Honkila M et al. *JAMA Network Open.*
