# Supplementary material for: Effect of Topical Antibiotics on Duration of Acute Infective Conjunctivitis in Children: A Randomized Clinical Trial and a Systematic Review and Meta-analysis
Source: JAMA Netw Open. 2022 Oct 4;5(10):e2234459. doi: 10.1001/jamanetworkopen.2022.34459 (PMC9533187; doi:10.1001/jamanetworkopen.2022.34459)
Supplement: Supplement 3. — Data Sharing Statement [file jamanetwopen-e2234459-s003.pdf]

Data Sharing Statement

Honkila. Effect of Topical Antibiotics on Duration of Acute Infective Conjunctivitis in Children. *JAMA Netw Open*. Published October 04, 2022. doi:10.1001/jamanetworkopen.2022.34459

Data
Data available: No
